# The changing molecular epidemiology of HIV-1 in Liangshan prefecture, China, in 2021–2023

**DOI:** 10.3389/fmicb.2025.1520864

**Published:** 2025-03-17

**Authors:** Rong Pei, Ling Su, Chunnong Jike, Gang Yu, Ju Wang, Lin Xiao, Yubing Wang, Maogang Shen, Chang Zhou, Jiayi Liao, Yulian Zhang, Yifei Zheng, Joris Hemelaar

**Affiliations:** ^1^Nuffield Department of Population Health, Infectious Disease Epidemiology Unit, National Perinatal Epidemiology Unit, University of Oxford, Oxford, United Kingdom; ^2^School of Public Health, Chengdu University of Traditional Chinese Medicine, Chengdu, China; ^3^Sichuan Provincial Center for Disease Control and Prevention, Center for AIDS/STD Control and Prevention, Chengdu, China; ^4^Liangshan Prefecture Centre for Disease Control and Prevention, Xichang, Xichang, China

**Keywords:** HIV-1, molecular epidemiology, circulating recombinant forms, subtype, unique recombinant forms

## Abstract

**Introduction:**

Liangshan Prefecture is one of the areas in China most severely affected by human immunodeficiency virus (HIV), but little is known about the molecular epidemiology of HIV-1 in this area. We aimed to analyze the distribution of HIV-1 genetic variants in Liangshan Prefecture in recent years.

**Methods:**

8,523 blood samples were collected from people living with HIV with treatment failure and newly diagnosed individuals in all 17 counties and cities in Liangshan Prefecture between 2021 and 2023.

**Results:**

The majority of study participants were male (66%), farmers (78%) and illiterate (53%). The main HIV-1 transmission routes were heterosexual contact (57%) and injecting drug use (27%). Among the 6,298 successfully obtained *pol* sequences the following HIV-1 variants were identified: CRF07_BC (93.9%), CRF08_BC (3.3%), CRF01_AE (1.4%), URFs (0.9%), CRF105_0108 (0.1%), CRF55_01B (0.1%), subtype B (0.1%), subtype C (0.1%), CRF88_BC (0.1%), CRF83_cpx (0.1%), CRF85_BC (0.03%), CRF67_01B (0.02%), CRF77_cpx (0.02%), and subtype A (0.02%). During the study period, the proportion of CRF07_BC gradually decreased, while other HIV-1 variants increased, a shift seen across all counties in Liangshan Prefecture. Newly diagnosed patients mainly acquired HIV through heterosexual transmission (86.7%), had a lower proportion of CRF07_BC (90.9%) and higher proportion of other HIV-1 variants, compared to treatment failure patients.

**Conclusion:**

Future prevention and control policies need to take these changes into account.

## 1 Introduction

The number of people living with HIV (PLWH) worldwide in 2023 was 39.9 million (WHO, 2024) and AIDS claimed a life every minute (UNAIDS, 2024). HIV/AIDS remains a major public health problem and a major cause of death in China (He, 2021) and the HIV epidemic in China accounts for 3% of the global HIV prevalence (Vrancken et al., 2020). At the end of 2023, there were 1,289,700 PLWH in China (China Center for Disease Control and Prevention, 2024). The HIV epidemic in China is unevenly distributed, and the annual number of newly diagnosed HIV cases reported as well as the total number of PLWH in Sichuan Province are the highest among provinces in China for several successive years (Zhou et al., 2022; Yang et al., 2023). Liangshan Prefecture within Sichuan Province, an autonomous prefecture with the largest population of Yi people in Southwest China, contains the largest proportion of PLWH in Sichuan Province. By the end of 2023, the number of PLWH in Liangshan Prefecture accounted for 3.7% of the national total. Within Liangshan Prefecture, prevalence rates of HIV are over 1% in Butuo County, Zhaojue County, Meigu county, Yuexi County, and Jinyang County, which are the counties with the highest HIV prevalence in China (Yuan et al., 2020b).

Global and regional HIV-1 genetic diversity is complex and evolving and poses a major challenge to HIV prevention and treatment. The global proportion of HIV-1 recombinants has consistently increased over time (Hemelaar et al., 2019; Nchinda et al., 2023). In China, the distribution of HIV-1 subtypes and Circulating Recombinant Forms (CRFs) is highly diverse and complex, and HIV-1 variant distribution varies significantly between regions (He, 2021). As the number of PLWH rapidly increases, the molecular epidemiology of HIV-1 in China is undergoing great changes (Zhong, 2019; Fan et al., 2022). More than 30 HIV-1 variants have been identified, with CRF01_AE, CRF07_BC, subtype B, Unique Recombinant Forms (URFs), CRF55_01B, CRF08_BC, and subtype C the predominant HIV-1 variants circulating in China (Vrancken et al., 2020; Ye et al., 2022). In the 1980s, CRF07_BC was originally reported in Yunnan Province and spread quickly among injecting drug users (IDUs). In recent years, CRF07_BC has been introduced in men who have sex with men (MSM) populations, which drove its spread to other parts of China (Vrancken et al., 2020). In Liangshan Prefecture CRF07_BC was the predominant strain in IDUs, with high genetic diversity when compared to strains prevalent in other provinces and cities in China (Li et al., 2015).

Liangshan Prefecture is located on the key route through which drugs from the “Golden Triangle” flow into mainland China, and injecting drug use was the main route of HIV transmission in this area in the past (Ruan et al., 2004; Ruan et al., 2005). Several studies reported that HIV infected IDUs in Liangshan Prefecture could serve as a source of HIV transmission to other regions of China (Meng et al., 2013; Li et al., 2015). However, sexual transmission became the predominant mode of HIV transmission in Liangshan Prefecture from 2014 (Wang et al., 2017). The genotype of HIV-1 is related to the route of transmission, and there are differences in epidemic scale and distribution characteristics of HIV-1 variants (Hemelaar, 2013; Nchinda et al., 2023). Understanding the trends in genotypes among newly diagnosed HIV infections following shifts in transmission routes can effectively enhance targeted intervention efforts. Existing research on HIV-1 variant distribution in Liangshan Prefecture is limited to drug resistance studies in specific counties or small population groups (Yuan et al., 2020a; Yuan et al., 2020b; Cao et al., 2023; Yuan et al., 2024). However, there are currently no studies on the molecular epidemiology and genotype trends in Liangshan Prefecture as a whole. We therefore aimed to analyze the changing molecular epidemiology of HIV-1 in Liangshan Prefecture in 2021–2023.

## 2 Materials and methods

### 2.1 Study population and sample collection

**Figure 1:**
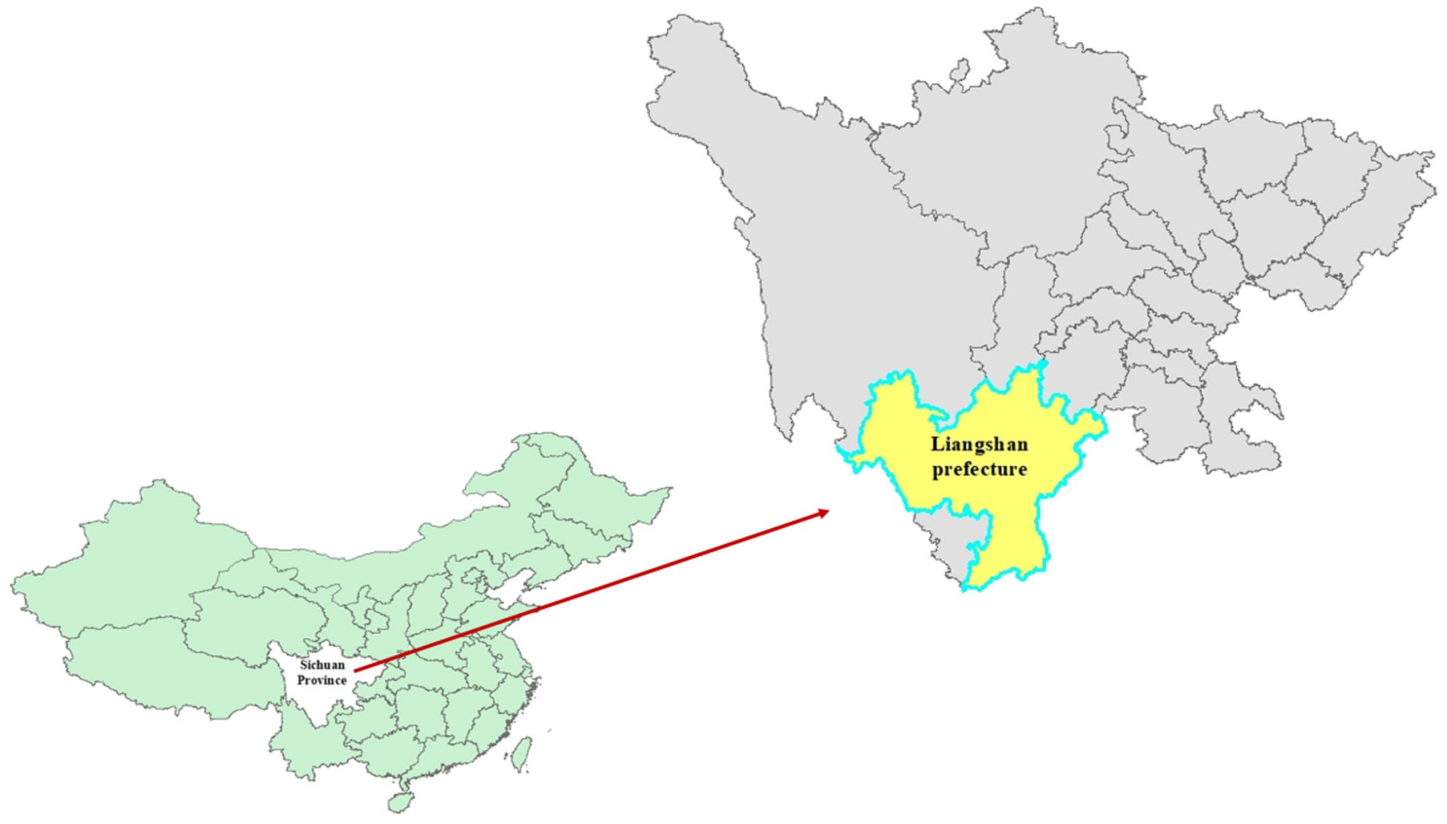
The geographical location of Liangshan Prefecture within Sichuan Province in China.

Liangshan Prefecture is located in the southwestern mountainous area of Sichuan Province (Figure 1). The area primarily depends on agriculture and transportation infrastructure is basic, resulting in a relatively low level of economic development. Blood samples were collected for testing from all 15 counties and two cities in Liangshan Prefecture between 2021 and 2023. The samples included all individuals who experienced treatment failure (defined as having viral load ≥1,000 copies/ml or being assessed by physicians as having poor treatment outcomes) as well as newly diagnosed individuals. Liangshan Prefecture conducted comprehensive HIV testing for the entire population in 2018 and 2022. Every year, various channels are used to promote and encourage the public to undergo HIV testing, resulting in a very high overall testing rate. Therefore, it is highly likely that newly diagnosed cases represent new infections and reflect the characteristics of recent transmissions.

Plasma samples were collected in accordance with standard procedures (Xing et al., 2015) by laboratory personnel of the local Center for Disease Control and Prevention (CDC) and were transported to the Sichuan Provincial Center for Disease Control and Prevention for HIV-1 drug resistance testing (Su et al., 2018).

Patients’ demographic information - including sex, age, marital status, ethnicity, education level, and transmission route - was collected from the National HIV/AIDS Comprehensive Response Information Management System, a web-based real-time database managed by the National Center for AIDS/STD Control and Prevention (NCAIDS) of the Chinese Center for Disease Control and Prevention (CDC) (Mao et al., 2010).

All patients voluntarily participated in the study and signed informed consent forms before enrolment. The study protocol was approved by the Ethics Committee of the Chengdu University of TCM. All research methods in this study were carried out according to the approved guidelines.

### 2.2 HIV-1 RNA extraction, amplification, and sequencing

Viral nucleic acid was obtained from 200 μL plasma of PLWH by extraction machines (MagNA Pure LC system, Roche, Branchburg, NJ). HIV-1 *pol* sequences were amplified and sequenced. Reverse Transcription-Polymerase Chain Reaction (RT-PCR) was used to amplify the full-length protease gene and the first 300 codons of the reverse transcriptase gene. Two rounds of PCR amplification were used, in accordance with the HIV-1 Genotype Drug Resistance Detection and Quality Assurance Guidelines (2013 Edition) (Su et al., 2016; Zhou et al., 2022). The PCR products were electrophoresed in 1% agarose gel, and the amplified PCR products were purified and bulk sequenced by Beijing Genomics Research Center Ltd. A total of 6,298 HIV-1 *pol* sequences covering 1,060 base pairs (HXB2: 2,253-3,312) were successfully obtained.

### 2.3 HIV-1 genotyping

The HIV sequences were aligned with reference sequences from the Los Alamos National Laboratory’s HIV-1 database^1^ using MEGA version 7.0, with subsequent minor manual adjustments for accuracy. To determine the genotype of each sequence, we conducted phylogenetic analysis with MEGA, estimating a maximum likelihood tree for the *pol* sequences using the GTR + G + I nucleotide substitution model. Bootstrap resampling (1,000 iterations) was used to construct the phylogenetic tree, ensuring statistical confidence in clade definitions. We visualized the resulting phylogenetic trees using FigTree v1.4.3.^2^ Preliminary genotype assignments were made by clustering sample sequences with international reference strains on the tree. These findings were then reviewed and validated using the online HIV Databases BLAST tool.^3^

### 2.4 Statistical analysis

Demographic characteristics of the study participants were summarized using frequencies and percentages. Chi-squared test was used to compare the distributions of the various HIV-1 variants according to sampling and participant characteristics. Significance was established at *p* < 0.05. Mapping and visualization were accomplished using ArcGIS 10.2 software. IBM SPSS 22 software was utilized for statistical analysis.

## 3 Results

### 3.1 Participant characteristics

A total of 8,523 blood samples were collected from 2021 to 2023. Among these, 304 samples lacked demographic information, leaving 8,219 valid samples. A total of 6,298 *pol* region sequences (76.6%) were successfully obtained following nucleic acid amplification and gene sequencing. 1,999 (31.7%) newly diagnosed and 4,299 (68.3%) treatment failure cases were included in our analyses. Characteristics of participants are summarized in Table 1. Of the 6,298 participants in this study, the majority were male (4,158; 66.0%). 3,129 (49.7%) participants were married or cohabiting and 3,343 (53.1%) were illiterate. 4,914 (78%) patients were farmers and participants aged 25–44 years accounted for the highest proportion (3,770; 59.8%). The most common mode of HIV-1 transmission was heterosexual contact (3,592, 57.0%), followed by injecting drug use (1,717, 27.3%). Most of the participants were of Yi ethnicity (5,654, 89.8%). The largest number of samples was collected in 2021 (2,996, 47.6%), followed by 2022 (1,847, 29.3%) and 2023 (1,455, 23.1%).

**Table 1:** Characteristics of people living with HIV in Liangshan Prefecture, China, in 2021–2023.

Variables	Total *n* (%)	New diagnosis *n* (%)	Treatment failure *n* (%)
Total	6,298 (100)	1999 (31.7)	4,299 (68.3)
Year of sample collection			
2021	2,996 (47.6)	951 (31.0)	2063 (48.0)
2022	1847 (29.3)	82 (2.7)	1769 (41.1)
2023	1,455 (23.1)	2023 (66.3)	467 (10.9)
Time since diagnosis (years)			
New diagnosis	1999 (31.7)		
1–5	2054 (32.6)		
6–10	1,275 (20.3)		
>10	970 (15.4)		
Gender			
Male	4,158 (66.0)	1,070 (53.5)	3,088 (71.8)
Female	2,140 (34.0)	929 (46.5)	1,211 (28.2)
Age (years)			
<18	688 (10.9)	207 (10.4)	481 (11.2)
18–24	339 (5.4)	178 (8.9)	161 (3.7)
25–44	3,770 (59.8)	1,011 (50.6)	2,759 (64.2)
45–64	1,287 (20.5)	501 (25.1)	786 (18.3)
≥65	214 (3.4)	102 (5.1)	112 (2.6)
Ethnicity			
Yi	5,654 (89.8)	1705 (85.3)	3,949 (91.9)
Han	605 (9.6)	271 (13.6)	334 (7.8)
Others	39 (0.6)	23 (1.2)	16 (0.4)
Transmission route			
Heterosexual	3,592 (57.0)	1734 (86.7)	1858 (43.2)
Injecting drug use	1717 (27.3)	37 (1.9)	1,680 (39.1)
Mother to child transmission	459 (7.3)	83 (4.2)	376 (8.7)
Men who have sex with men	35 (0.6)	13 (0.7)	22 (0.5)
Heterosexual +Injecting drug use	211 (3.4)	12 (0.6)	199 (4.6)
Uncertain	284 (4.5)	120 (6.0)	164 (3.8)
Education			
Illiterate	3,343 (53.1)	917 (45.9)	2,426 (56.4)
Primary school	2,242 (35.6)	776 (38.8)	1,466 (34.1)
Junior high school	517 (8.2)	204 (10.2)	313 (7.3)
High school or technical secondary school	128 (2.0)	52 (2.6)	76 (1.8)
College or above	68 (1.1)	50 (2.5)	18 (0.4)
Marital status			
Married or cohabiting	3,129 (49.7)	870 (43.5)	2,259 (52.5)
Unmarried	2030 (32.2)	568 (28.4)	1,462 (34.0)
Divorced or widowed	1,074 (17.1)	556 (27.8)	518 (12.0)
Unknown	65 (1.0)	5 (0.3)	60 (1.4)
Occupation			
Farmers	4,914 (78.0)	1,674 (83.7)	3,240 (75.4)
Laborer	100 (1.6)	25 (1.3)	75 (1.7)
Unemployed	250 (4.0)	36 (1.8)	214 (5.0)
Government and public institutions	39 (0.6)	31 (1.6)	8 (0.2)
Business Services	23 (0.4)	10 (0.5)	13 (0.3)
Children and students	700 (11.1)	189 (9.5)	511 (11.9)
Others	272 (4.3)	34 (1.7)	238 (5.5)
CD4+ T lymphocyte count (pcs/μL)			
<200	958 (15.2)	218 (10.9)	740 (17.2)
200–350	1756 (27.9)	511 (25.6)	1,245 (29.0)
>350	3,429 (54.4)	1,162 (58.1)	2,267 (52.7)
Missing	155 (2.5)	108 (5.4)	47 (1.1)

The majority of patients were male (53.5%) among newly diagnosed patients in the study, which was a lower proportion than among the treatment failure cases (71.8%). Participants aged 25–44 years accounted for the highest proportion (50.6%), which was lower than among treatment failure cases (64.2%), while the proportion of patients aged 18–24 (8.9%) was higher than among treatment failure cases (3.7%). 10.9% of newly diagnosed patients and 17.2% of treatment failure patients had a CD4+ T lymphocyte count <200 pcs/μl. The majority of newly diagnosed cases were due to heterosexual contact (86.7%). In contrast, treatment failure patients are primarily associated with heterosexual contact (43.2%) and injecting drug use (39.1%). 45.9% of newly diagnosed patients were illiterate, which is lower than among treatment failure cases (56.4%). Most newly diagnosed patients were farmers (83.7%), which is higher than among treatment failure cases (75.4%).

### 3.2 HIV-1 variant distribution

**Figure 2:**
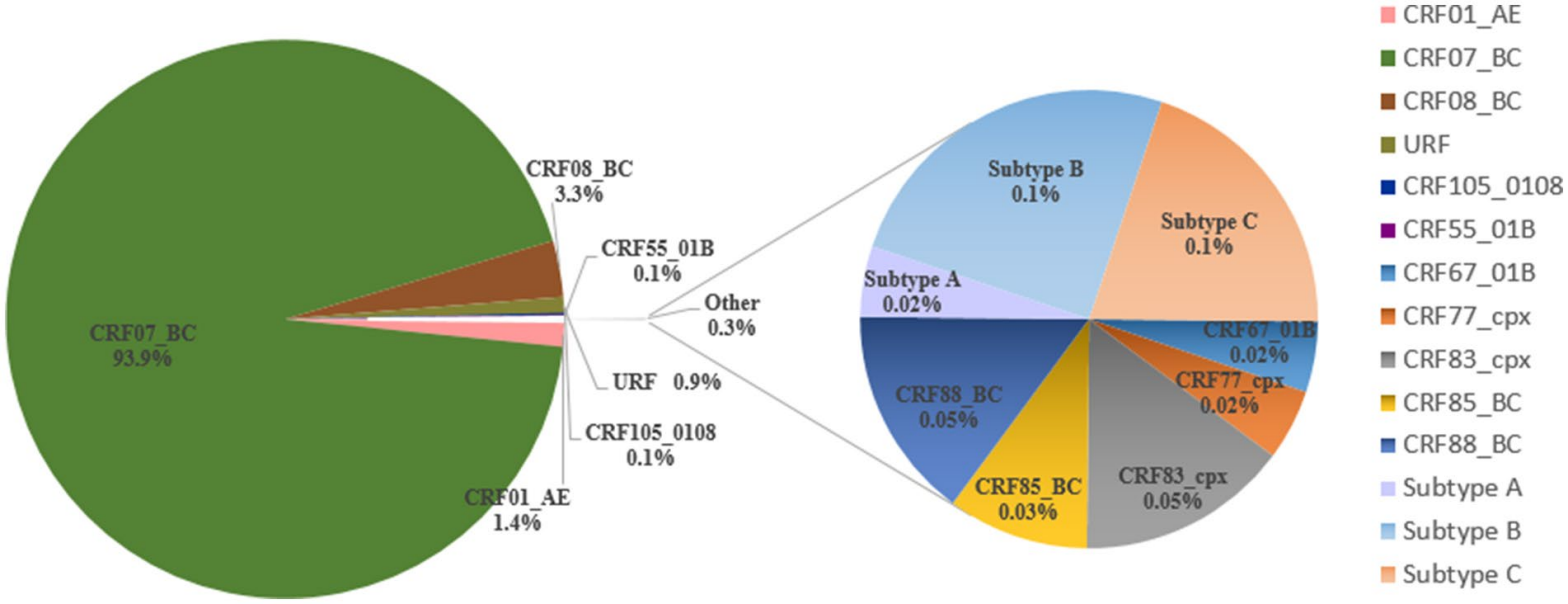
Distribution of HIV-1 variants among people living with HIV in Liangshan Prefecture, China, in 2021–2023.

A total of three HIV-1 subtypes, 10 CRFs, and URFs were identified among the 6,298 successfully obtained *pol* sequences. The distribution of these variants is depicted in Figure 2. CRF07_BC (93.9%) was the most prevalent HIV-1 variant, followed by CRF08_BC (3.3%), CRF01_AE (1.4%), and URFs (0.9%). Other variants found were CRF105_0108 (0.1%), CRF55_01B (0.1%), subtype B (0.1%), subtype C (0.1%), CRF88_BC (0.1%), CRF83_cpx (0.1%), CRF85_BC (0.03%), CRF67_01B (0.02%), CRF77_cpx (0.02%), and subtype A (0.02%).

The characteristics of HIV-1 variants in Liangshan Prefecture are shown in Table 2. The distribution of HIV-1 variants was significantly different (*p* < 0.05) according to year of sample collection, time since diagnosis, patient category, route of transmission and ethnicity. The HIV-1 variants among newly diagnosed patients were more diverse, with a decreasing proportion of CRF07_BC (90.9%), compared to treatment failure patients (95.3%). Compared to other transmission routes, the CRF07_BC proportion of the patients infected through heterosexual transmission is lower and the HIV-1 variants are more diverse. The proportion of CRF07_BC is higher among the Yi ethnic group compared to other ethnic groups.

**Table 2:** Characteristics of HIV-1 variants among people living with HIV in Liangshan Prefecture, China, in 2021–2023.

Variables	CRF01_AE *n* (%)	CRF07_BC *n* (%)	CRF08_BC *n* (%)	CRF55_01B *n* (%)	CRF105_0108 *n* (%)	URFs *n* (%)	Minor *n* (%)	Total *n*	*p*- value
Total	89 (1.4)	5,914 (93.9)	205 (3.3)	6 (0.1)	7 (0.1)	57 (0.9)	20 (0.3)	6,298	
Year of sample collection									<0.001
2021	36 (1.2)	2,838 (94.7)	91 (3.0)	2 (0.1)	0	20 (0.7)	9 (0.3)	2,996	
2022	14 (0.8)	1739 (94.2)	65 (3.5)	3 (0.2)	1 (0.1)	19 (1.0)	6 (0.3)	1847	
2023	39 (2.7)	1,337 (91.9)	49 (3.4)	1 (0.1)	6 (0.4)	18 (1.2)	5 (0.3)	1,455	
Time since diagnosis (years)									<0.001
New diagnosis	50 (2.5)	1817 (90.9)	80 (4.0)	5 (0.3)	5 (0.3)	31 (1.6)	11 (0.6)	1999	
1–5	22 (1.1)	1946 (94.7)	64 (3.1)	1 (0.05)	2 (0.1)	14 (0.7)	5 (0.2)	2054	
6–10	13 (1.0)	1,224 (96.0)	30 (2.4)	0	0	4 (0.3)	4 (0.3)	1,275	
>10	4 (0.4)	927 (95.6)	31 (3.2)	0	0	8 (0.8)	0	970	
Patient category									<0.001
New diagnosis	50 (2.5)	1817 (90.9)	80 (4.0)	5 (0.3)	5 (0.3)	31 (1.6)	11 (0.6)	1999	
Treatment failure	39 (0.9)	4,097 (95.3)	125 (2.9)	1 (0.05)	2 (0.1)	26 (0.6)	9 (0.2)	4,299	
Transmission route									<0.001
Injecting drug use	13 (0.8)	1,637 (95.3)	56 (3.3)	0	0	10 (0.6)	1 (0.1)	1717	
Heterosexual	67 (1.9)	3,227 (92.6)	131 (3.6)	4 (0.1)	7 (0.2)	39 (1.1)	17 (0.5)	3,592	
Mother to child transmission	1 (0.2)	446 (97.2)	6 (1.3)	0	0	4 (0.9)	2 (0.4)	459	
Men who have sex with men	3 (8.6)	30 (85.7)	0	2 (5.7)	0	0	0	35	
Heterosexual+Injecting drug use	1 (0.5)	202 (95.7)	6 (2.8)	0	0	2 (0.9)	0	211	
Uncertain	4 (1.4)	272 (95.8)	6 (2.1)	0	0	2 (0.7)	0	284	
Ethnicity									<0.001
Yi	62 (1.1)	5,358 (94.8)	171 (3.0)	2 (0.05)	1	48 (0.8)	12 (0.2)	5,654	
Han	26 (4.3)	523 (86.4)	29 (4.8)	4 (0.7)	6 (1.0)	9 (1.5)	8 (1.3)	605	
Others	1 (2.6)	33 (84.6)	5 (12.8)	0	0	0	0	39	

### 3.3 Relationship between HIV-1 variants and transmission routes

**Figure 3:**
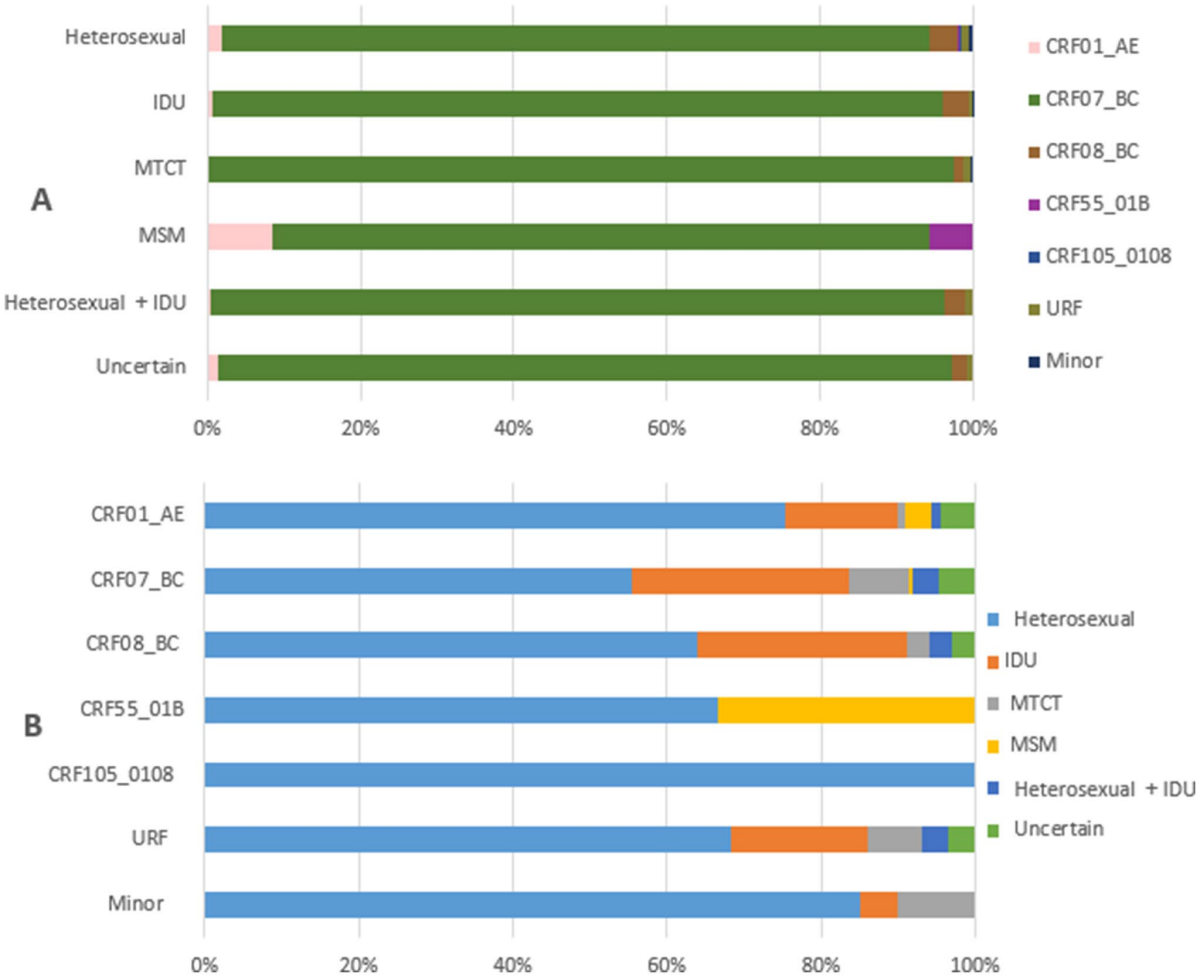
Relationship between transmission routes and HIV-1 variants among people living with HIV in Liangshan Prefecture, China, in 2021–2023. **(A)** The distribution of HIV-1 variants by transmission routes among people living with HIV in Liangshan Prefecture, China, in 2021–2023. **(B)** The distribution of transmission routes for HIV-1 variants among people living with HIV in Liangshan Prefecture, China, in 2021–2023. IDU, injecting drug user; MSM, men who have sex with men; MTCT, mother to child transmission; URF, unique recombinant form.

Figure 3 illustrates the relationship between HIV-1 variants and transmission routes among PLWH in Liangshan Prefecture in 2021–2023. For sexual transmission routes, both MSM and heterosexual, CRF07_BC accounts for a smaller proportion and more diverse HIV-1 variants were found, compared to other transmission routes (Figure 3A). In contrast, mother-to-child transmission (MTCT) is almost exclusively associated with CRF07_BC, similar to transmission through injecting drug use. The MSM transmission route involves only three variants, CRF01_AE, CRF07_BC and CRF55_01B, and its proportion of CRF07_BC is lower than the other routes. CRF105_0108 was only found in heterosexual transmission cases, while CRF55_01B was only present in MSM and heterosexual transmission cases (Figure 3B).

**Figure 4:**
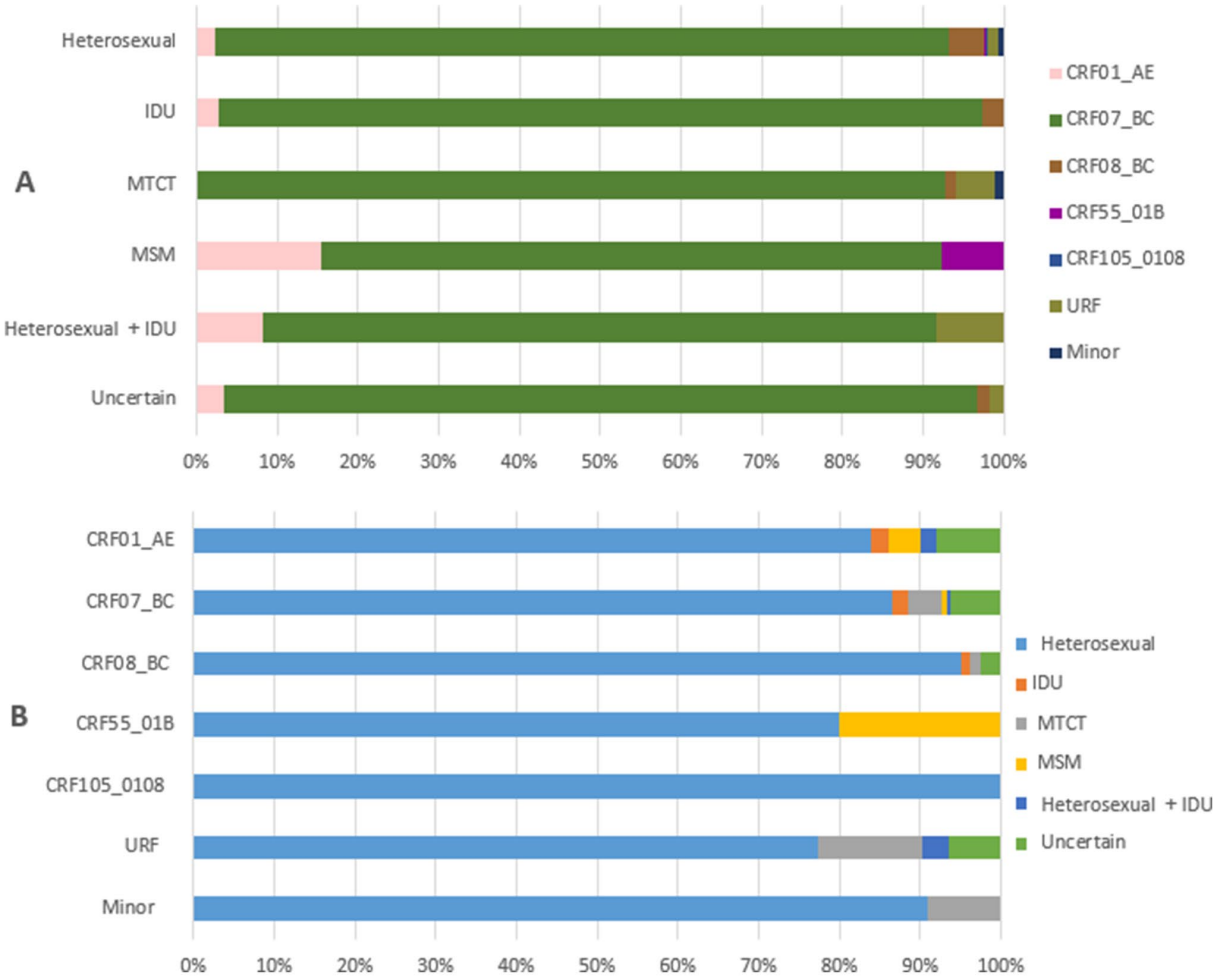
Relationship between transmission routes and HIV-1 variants among newly diagnosed people living with HIV in Liangshan Prefecture, China, in 2021–2023. **(A)** The distribution of HIV-1 variants by transmission routes among newly diagnosed patients in Liangshan Prefecture, China, in 2021–2023. **(B)** The distribution of transmission routes for HIV-1 variants among newly diagnosed patients in Liangshan Prefecture, China, in 2021–2023. IDU, injecting drug user; MSM, men who have sex with men; MTCT, mother to child transmission; URF, unique recombinant form.

The relationship between HIV-1 variants and transmission routes among newly diagnosed patients in Liangshan Prefecture is shown in Figure 4. Among newly diagnosed patients the heterosexual transmission route was the most dominant route for all HIV-1 variants (Figure 4B). On the other hand, all of the transmission routes among newly diagnosed patients were associated with more diverse HIV-1 variants compared to all PLWH (Figures 3A, 4A).

### 3.4 Regional distribution of HIV-1 variants

**Figure 5:**
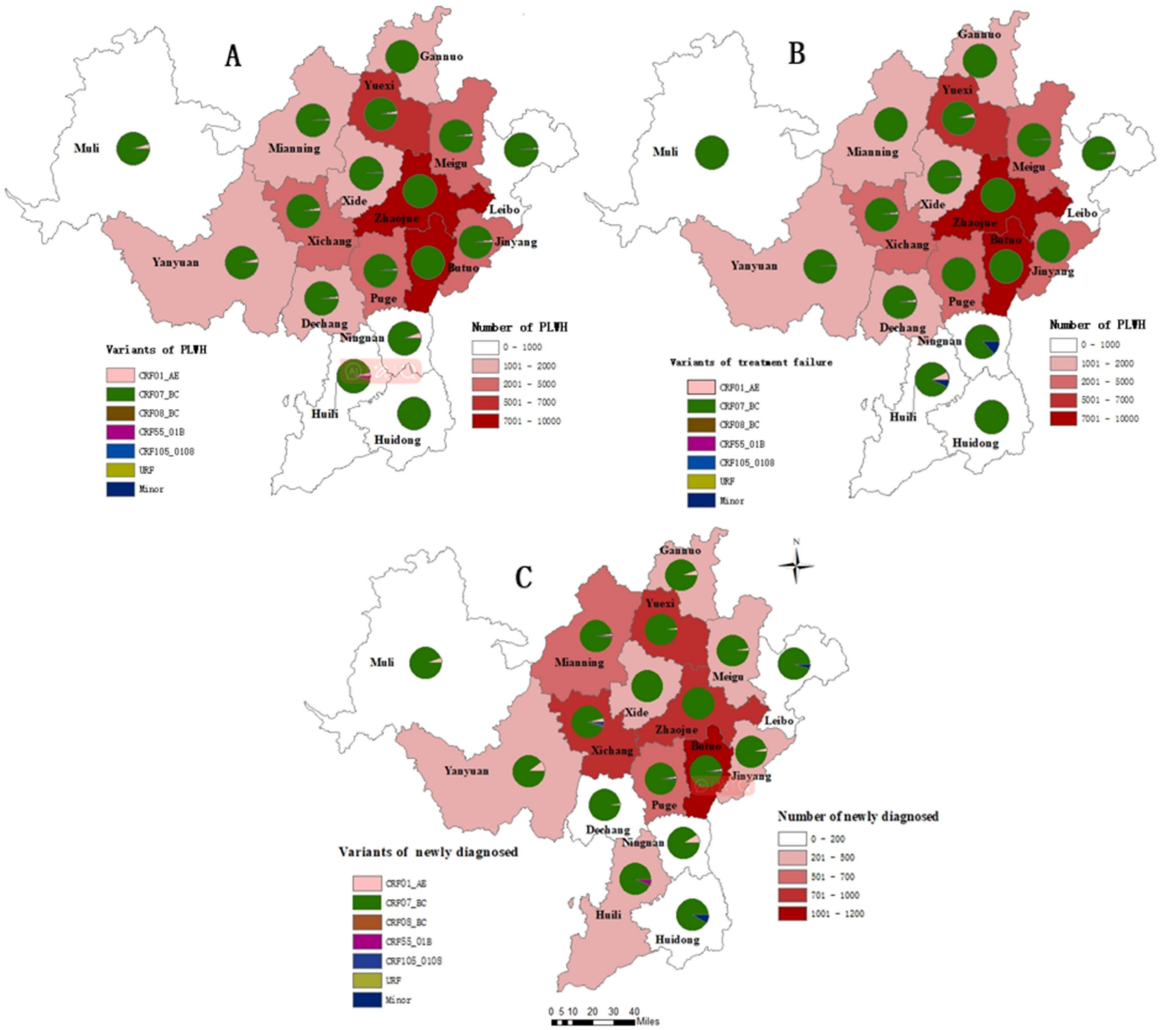
Regional distribution of HIV-1 variants among people living with HIV in Liangshan Prefecture, China, in 2021–2023. **(A)** Regional distribution of HIV-1 variants among all people living with HIV in Liangshan Prefecture, China, in 2021–2023. **(B)** Regional distribution of HIV-1 variants among treatment failure patients in Liangshan Prefecture, China, in 2021–2023. **(C)** Regional distribution of HIV-1 variants among newly diagnosed patients in Liangshan Prefecture, China, in 2021–2023.

The regional distribution of the HIV-1 variants across different counties in Liangshan Prefecture in 2021–2023 is shown in Figure 5. It is evident that the predominant HIV-1 variant in all counties is CRF07_BC, particularly in Zhaojue, Butuo, Huidong and Ganluo counties (Figure 5A). The HIV-1 variant distribution among treatment failure patients shows that more regions tend to have an absolute predominance of CRF07_BC (Figure 5B). Across all regions among newly diagnosed patients, the proportion of CRF07_BC was lower and other variants were more prevalent, compared to treatment failure patients (Figure 5C).

## 4 Discussion

This is the first study to report the distribution and trends of HIV-1 variants in Liangshan Prefecture, with samples of newly diagnosed HIV infections and treatment failure patients from 2021 to 2023. A total of three HIV-1 subtypes, 10 distinct CRFs, and URFs were identified, with CRF07_BC being the predominant variant. During the study period, the proportion of CRF07_BC gradually decreased, while other HIV-1 variants increased, a shift seen across all counties in Liangshan Prefecture. Newly diagnosed patients mainly acquired HIV through heterosexual transmission, had a lower proportion of CRF07_BC and higher proportion of other HIV-1 variants.

Male patients account for two-thirds of the total number of HIV infections, consistent with other studies conducted in China (Lu et al., 2021; Ye et al., 2022; Zhou et al., 2022). Over the years, there has been a steady increase in the number of HIV infections among females, with heterosexual transmission being the primary route. This trend has brought the disease into the general population, posing a significant challenge to HIV/AIDS prevention and control efforts in China (He, 2021). Although Liangshan Prefecture remains the most severely affected area of the HIV epidemic in China (Yuan et al., 2020b; Yuan et al., 2024), the number of new cases has gradually decreased over the past 5 years with the implementation of a series of prevention and control measures, such as free condoms, free HIV tests, health education, free treatment and expanded HIV tests. Our study further confirms that heterosexual transmission remains the predominant route of infection, particularly among newly diagnosed cases in Liangshan Prefecture. The study shows that more than half of the HIV infected individuals in Liangshan Prefecture are illiterate, indicating a significantly lower level of education compared to PLWH in other parts of China (Lu et al., 2021; Fan et al., 2022). The education level improved in the newly diagnosed cases and was better than in previous local studies (Liu et al., 2020; Yuan et al., 2024). A lower level of education may lead to a lack of health-related knowledge, which increases the likelihood of engaging in high-risk behaviors. Therefore, targeted health education should be prioritized for those with limited educational backgrounds. Individuals aged 25 to 44 account for the highest proportion of HIV infections, also among newly diagnosed cases, with most cases resulting from heterosexual transmission. This age group is sexually active and represents a key population to target for HIV prevention efforts.

CRF07_BC is the predominant variant in Liangshan Prefecture, with a further three HIV-1 subtypes, nine CRFs and URFs identified. This distribution differs significantly from the nationwide pattern, where CRF01_AE is predominant, and CRF07_BC accounts for approximately 20% of HIV infections (He, 2021; Ye et al., 2022). The high proportion of CRF07_BC may be attributed to its initial transmission primarily among IDUs and heterosexual individuals (Gao et al., 2024), as these remain the main routes of infection in Liangshan Prefecture. However, CRF07_BC has also become a major HIV-1 variant among MSM (Ge et al., 2021; Pang et al., 2023). The proportion of new HIV diagnoses attributed to the MSM transmission route has exceeded 25% nationwide (National Disease Control and Prevention Administration, 2023). Therefore, efforts should be strengthened to prevent the further spread of CRF07_BC through homosexual transmission.

Our study revealed several noteworthy observations. First, CRF07_BC is the main HIV-1 variant in Liangshan Prefecture. Second, the proportion of CRF07_BC gradually decreased, while the other HIV-1 variants increased over time. Third, newly diagnosed patients mainly acquired HIV through heterosexual transmission, and are associated with lower proportion of CRF07_BC and more diverse HIV-1 variants. Lastly, the HIV-1 variant shift was seen across all counties and cities in Liangshan Prefecture. These trends in genotype distribution may be linked to shifts in transmission routes. As the number of newly diagnosed infections through heterosexual transmission increases, the diversity of genotypes also broadens. Previous studies have shown that CRF07_BC is associated with a lower viral load and a slower rate of disease progression (Huang et al., 2014; Cheng et al., 2022; Ye et al., 2022). However, with the evolving genotypic landscape among newly diagnosed individuals in Liangshan, the proportion of CRF07_BC is gradually decreasing and other diverse HIV-1 variants are increasing, suggesting a growing challenge for prevention and control efforts. In addition, factors such as economic growth and population mobility can impact the dynamics of the HIV/AIDS epidemic (Qin et al., 2017; Eshraghian et al., 2020). Liangshan Prefecture is undergoing rapid economic growth and has a significant migrant population. As a result, it is vital to closely monitor the trends of the HIV epidemic to develop targeted and effective prevention and control measures.

Our study had several strengths. First, a very large number of samples were collected from all counties and cities in Liangshan Prefecture. Second, data was collected in recent years. Third, most newly diagnosed cases likely represent new HIV infections due to comprehensive population HIV testing in Liangshan Prefecture in 2018 and 2022.

Our study also had some limitations. First, treatment failure patients do not represent all of the PLWH in Liangshan Prefecture, as PLWH with lower viral loads and successful treatment were not included in the genotype testing. Second, around a quarter of blood samples were not genotyped. However, our dataset includes data from all regions of Liangshan Prefecture, providing a representative overview of the overall situation. We anticipate making even stronger inferences about the characteristics of the transmission networks by improving the completeness of molecular surveillance data in the future.

## 5 Conclusion

In summary, analysis of the HIV-1 molecular epidemic in Liangshan Prefecture, China, in 2021–2023 has revealed three significant observations: First, CRF07_BC remains the predominant variant, and its proportion is gradually declining, while other HIV-1 variants rise over time. Second, newly diagnosed patients are mainly due to heterosexual transmission, and are associated with a lower proportion of CRF07_BC and more diverse HIV-1 variants. Third, the HIV-1 variant shift was seen across all counties and cities in Liangshan Prefecture. Future research should focus on exploring the complex interactions between viral genetics, host factors, and behavioral patterns to develop more targeted and effective HIV/AIDS prevention and control strategies.

## Data Availability

The original contributions presented in the study are included in the article/supplementary material, further inquiries can be directed to the corresponding author.

## Footnotes

^1^ http://www.hiv.lanl.gov

^2^ http://tree.bio.ed.ac.uk/software/figtree/

^3^ https://www.hiv.lanl.gov/content/index
